# Comparison of Polar Active Watch and Waist- and Wrist-Worn ActiGraph Accelerometers for Measuring Children’s Physical Activity Levels during Unstructured Afterschool Programs

**DOI:** 10.3390/ijerph15102268

**Published:** 2018-10-16

**Authors:** Youngdeok Kim, Marc Lochbaum

**Affiliations:** 1 Department of Kinesiology and Sport Management, Texas Tech University, Lubbock, TX 79409, USA; marc.lochbaum@ttu.edu; 2 The Academy of Education, Vytautas Magnus University, Kaunas LT-44248, Lithuania

**Keywords:** accelerometer, validity, youth, health

## Abstract

*Background*: The purpose of this study was to examine the convergent validity of the Polar Active Watch (PAW), a consumer-grade wrist-worn activity monitor, against waist- and wrist-worn research-grade monitors, the ActiGraph GT3X+/GT9X accelerometers, in children. *Methods*: Fifty-one children (18 boys; mean age = 10.30 ± 0.91 years) wore the three monitors (PAW, GT3X+, and GT9X) during an 80-min afterschool program across five school days. Time spent in sedentary, light-intensity (LPA), and moderate- and vigorous-intensity physical activity (MVPA) were estimated from each monitor. The correlation, mixed model, mean absolute percentage error, equivalence testing, and Bland-Altman analyses were used to examine the comparability of PA estimates of the PAW with GT3X+/GT9X accelerometers. *Results*: Moderate to strong correlations for sedentary and MVPA minutes, and weak correlation for LPA were observed between the PAW and GT3X+/GT9X accelerometers. Significant mean differences were found, where the PAW tended to overestimate time in sedentary and MVPA and underestimate LPA minutes, compared to the GT3X+/GT9X accelerometers. However, a non-significant mean difference in MVPA minutes was observed when using an adjusted MET threshold (≥4 METs) for the PAW, compared to the GT3X+ accelerometer. *Conclusions*: The PAW showed moderate convergent validity for sedentary and MVPA minutes against the GT3X+/GT9X accelerometers. However, caution is needed in the direct comparison between the monitors due to relatively large mean differences and within-group variability.

## 1. Introduction

Physical activity (PA) is one of the most important lifestyle behaviors associated with better health and wellbeing in all age groups. In particular, there is an extensive body of literature demonstrating positive health benefits of PA in children [1,2] such as a reduced risk for being overweight and obese [3], as well as better metabolic [4], bone [5], and mental health [6]. As a result, world and national public health authorities have released PA guidelines for children suggesting that school-aged children should engage in 60 min of daily moderate- and vigorous-intensity PA (MVPA) [7,8]. Further given the importance of school as a place for the promotion of PA in children, the Institute of Medicine recommends at least 30 min of MVPA per school day be accumulated in a school environment [9], which includes, but is not limited to, physical education, before and after-school PA programs, and recess [10]. 

A growing awareness of the critical role of PA in children’s health has emphasized the importance of tracking and quantifying the levels of their habitual PA [11]. A pedometer is a simple version of wearable PA monitor, and has also been advocated as a cost-effective intervention tool for the promotion of PA in various settings [12]. More recently, several manufacturers have produced consumer-grade wearable PA monitors [13] using advanced technology. According to a recent report from the International Data Corporation [14], the wearables market has expanded rapidly, where more than 102 million devices were shipped worldwide in 2016, which was approximately 20% higher than 2015. A majority of currently available monitors are equipped with modern accelerometry technology and use a proprietary algorithm to calculate different types of PA metrics, such as step counts, energy expenditure, PA intensity levels, etc. Advanced data transfer/management technologies have also been applied to display real-time PA data on various platforms, allowing end-users to interact with PA data in everyday life. These features provide the essential components of behavioral change techniques (e.g., self-monitoring, real-time feedback, goal setting) [15,16], and have shown to be promising for the promotion of PA in children [17,18].

As wearable PA monitors have increased in popularity, their measurement properties have become an ongoing topic of interest [19]. Specifically, the differences in hardware sensor and proprietary algorithm between brands and monitors may attribute to inconsistent and incomparable results. It has been reported that differences in PA estimates between activity monitors differently affect user’s perceived rewards for the same activity [20], which may negatively influence motivation to engage in PA [21], and be partly responsible for high levels of abandonment of activity monitors [20]. In addition, there is a growing number of studies utilizing consumer-grade activity monitors for research purposes, either as an assessment tool, intervention component, or both, where high levels of accuracy and comparability with other research-grade monitors are demanded [22]. Several studies have been conducted to address this issue by examining the validity and reliability of consumer-grade PA monitors for the assessment of different PA metrics [23,24,25]. However, the results are dependent on the types and brands of monitors, and are frequently inconsistent across settings [19]. Further, most importantly, a majority of studies have focused on the monitors produced by a few specific brands (e.g., Fitbit, Jawbone), requiring further validation studies for a monitor produced by a wide range of brands.

The Polar Active Watch (hereafter referred to as PAW) is one of the wrist-worn PA monitors produced by the Polar Electro Oy, Kimpel, Finland, particularly known for developing the first portable heart rate monitors in the 1970s. According to its brochure, the PAW is designed for the children in compliance with the required standards of the Consumer Product Safety Information Acyl/Children’s Product. The PAW is not primarily intended for research purposes; however, the unique ‘group solution’ features that allow end-users to manage and monitor multiple devices at the same time and graphically display the PA data via its companion web and mobile applications have been well adopted by researchers [26,27,28,29,30]. In particular, a recent study that examined the feasibility of PAW for the assessment of PA in children in free-living settings reported that the PAW was better accepted by children compared to other research-grade monitors, owing to its comfortable, waterproof watch-style design and digital display features [31], which are known to be associated with higher compliance levels in this population group [32].

Although the PAW is designed for children, the device has been previously validated for its accuracy in measuring energy expenditures among adults [33]. However, to the best of our knowledge, there are no studies examining the validity of PAW in children, one of the target end user groups of this device, particularly for the assessment of PA intensity levels. Therefore, this study sought to examine the validity of PAW in children based on the convergent method of assessment, which has been successfully applied for the validation of both consumer [34,35] and research-grade PA monitors [36,37]. The convergent validity refers to the extent to which the two PA monitors that are known to measure similar measurement constructs are positively correlated. Although the convergent validity does not directly imply the construct validity (i.e., degree to which the monitor measures what it is supposed to measure), it provides the form of evidence of construct validity, particularly when the counterpart monitor has known validity against a criterion measure [38,39]. In this regard, we compared PA intensity levels estimated from the PAW to the research-grade PA monitor, ActiGraph accelerometers, which have been previously validated in children [40,41] and frequently compared to different types of consumer-grade PA monitors [42,43,44]. Although the ActiGraph accelerometers have been extensively validated in the literature, the evidence is specific to the location of placement (wrist vs. waist), activity cut-points, as well as the model of ActiGgaph accelerometer used. Therefore, this study examined the convergent validity of PAW for the assessment different PA intensity levels in children, with particular focus on its comparability with wrist-worn GT3X+ and waist-worn GT9X ActiGraph accelerometers, using previously established cut-points for each wear location.

## 2. Materials and Methods

### 2.1. Study Setting and Participants

A total of 51 children (18 boys) aged between 9 and 11 years (mean age = 10.30 years old) participated in this study. All children were enrolled in a school-based afterschool program in a US public elementary school located in a high-poverty area in a mid-size west Texas city. The afterschool program, established as part of the US federally funded East Lubbock Promise Neighborhood (ELPN) project, offers a variety of sessions that include but are not limited to PA, arts, music, and science every school day. Each child had an individualized schedule requiring participation in two sessions per day of 40 min each, one of which was a mandatory PA session on every school day. The PA session was designed to promote moderate- and vigorous-intensity PA (MVPA) through age-appropriate and task-oriented activities (e.g., kicking, running, throwing), and taught by trained undergraduate and graduate level coaches in the school gym or on the playground. Other non-PA sessions were delivered in a classroom, auditorium room, or technology/science lab, where children were supervised and taught by trained afterschool teachers in a relaxed environment. Thus, the afterschool program covered the range of activity levels, from sedentary to vigorous intensity, in supervised school environments. Children participating in the afterschool program were healthy, without physical impairments that would limit their physical activities. Each child’s standing height (cm) and body weight (kg) were measured by trained staff using a stadiometer (SECA, Seca Co., Hamburg, Germany) and mechanical scale (Health-O-Meter Professional, Subbeam Products Co., Boca Raton, FL, USA). Participant characteristics are presented in Table 1. This study was conducted under the ELPN protocols approved by the Institutional Review Board of Texas Tech University (#503995), in accordance with the Declaration of Helsinki, and the parents’ informed consent and child’s verbal assent were obtained at the beginning of the semester. Interested readers can refer to a previous report [45] for more details about the ELPN project.

### 2.2. Procedures

PA data were collected using three accelerometers (PAW, waist-worn GT3X+, and wrist-worn GT9X accelerometers) during 80 min of afterschool programs for up to five school days for each child. Children were asked to visit a pre-designated classroom before the start of their first afterschool program session each day. Children were instructed to wear PAWs and GT9X accelerometers in the same order on their non-dominant wrists, and GT3X+ accelerometers on elastic belts around their waists through the end of the afterschool program in a day. The choice of wear location for the respective ActiGraph accelerometer was solely based on the discretion of researchers, given the size of accelerometers relative to the children’s wrist (i.e., GT9X < GT3X+), as well as available resources (i.e., wristbands and elastic belts).

### 2.3. Polar Active Watch

The PAW is a lightweight (45 g), watch-style uniaxial accelerometer, with a screen on its face displaying time and activity levels. The child’s sex, date of birth, height, and weight were entered to the Polar GOFIT website (https://polargofit.com/), and the Polar Websync Software (version 2.8.3, Polar Electro Oy, Kimpel, Finland) was used to initialize the device and transfer data from the device for further analyses. The PAW provides 30-s epoch metabolic equivalent task (MET) scores estimated by the manufacturer’s proprietary algorithm.

### 2.4. ActiGraph GT3X+ and GT9X Accelerometers

The GT3X+ (firmware v3.2.1, ActiGraph Inc, Pensacola, FL, USA) and GT9X (firmware v1.7.1) are two of the latest generations of ActiGraph accelerometers, that can be worn both at the waist or on the wrist using an elastic belt or watch band. Both devices record accelerations in three axes (vertical, antero-posterior, and medio-lateral), with a dynamic range of ±6 g for the GT3X+ and ±8 g for the GT9X, at sampling frequencies ranging from 30–100 Hz, which are converted into activity counts at a user-defined epoch length. For this study, the devices were set to collect data at 30 Hz, and the data were downloaded at 30-s epoch using the ActiGraph ActiLife software (version 6.13.3, ActiGraph Inc, Pensacola, FL, USA). All devices were synchronized to the time on the computer clock.

Time spent in different PA intensity levels was estimated. For the PAW, three different sets of MET thresholds were used:

- MET thresholds#1: Sedentary (<2.0 METs); Light-intensity PA (LPA) (2.00–3.49 METs); and moderate- and vigorous-intensity PA (MVPA) (≥3.50 METs)
- MET thresholds#2: Sedentary (<1.5 METs); Light-intensity PA (LPA) (1.50–2.99 METs); and moderate- and vigorous-intensity PA (MVPA) (≥3.00 METs)
- MET thresholds#3: Sedentary (<2.0 METs); LPA (2.01–3.99 METs); and MVPA (≥4 METs)

The first set of MET thresholds (PAW#1) were Polar-defined MET thresholds in Polar Active software. The second set of MET thresholds (PAW#2) were standard MET thresholds widely used in the literature; yet these thresholds may not be appropriate for children when a child-specific resting metabolic rate (RMR) is not accounted [46,47]. Since it is currently unknown whether or not the Polar proprietary algorithm adjusts for higher RMR in children when estimating MET values, we created additional thresholds (PAW#3), which have been used as alternative MET thresholds for children [47], including the US national health surveillance [48].

As previously noted, several activity cut-points have been proposed for the waist- and wrist-worn ActiGraph accelerometers, yet no single cut-point is recognized as standard. For this study, we selected the two cut-points previously proposed for each of the waist- and wrist-worn ActiGraph accelerometers:

- Evenson’s cut-points (GT3X+-Evenson): Sedentary (≤50), LPA (51–1146), and MVPA (≥1147) using activity counts per 30-s from the vertical axis, according to Reference [40].
- Chandler’s cut-points (GT9X-Chandler): Sedentary (<805), LPA (805–2645), and MVPA (≥2646) using activity counts per 30-s from the vertical axis, according to Reference [49].

The Evenson’s cut-points were developed using the older generation of ActiGraph accelerometer (model 7164), which has different internal mechanisms compared to the GT3X+ accelerometer. Based on the suggestion of Cain et al. [50], we enabled the low frequency extension filter during post data processing to attenuate possible biases in applying the Evenson’s cut-points to the GT3X+ activity counts data. The Chandler’s cut-points were developed using the GT3X+ accelerometer; however, a recent study reported that activity counts estimated from the GT3X+ and GT9X accelerometers were comparable [51], regardless of activation of the low frequency extension filter [52]. Regarding consistency, the low frequency extension filter was also enabled when processing the GT9X accelerometer data. In addition, the Evenson’s and Chanlder’s cut-points that were originally calibrated in 15-s and 5-s epoch lengths, respectively, were converted to the 30-s cut-points by multiplying by 2 and 6, respectively.

Time spent in different PA intensity levels during afterschool periods was calculated for each monitoring day. The first and last 10 mins of the data were removed prior to the calculation to account for the time spent in distributing and returning the devices. There were 152 data points recorded across the entire sample (median monitoring days: 3 days; interquartile range: 2–4).

### 2.5. Statistical Analysis

We first created scatter plots by examining the linear relation between MET values per 30-s obtained from the PAW and activity counts per 30-s obtained from the GT3X+ and GT9X accelerometers, respectively, for each monitoring day. The purpose of this analysis was to examine the convergence between monitors that are not influenced by the choice of MET thresholds for the PAW, and activity counts cut-points for the GT3X+ and GT9X accelerometers. An average time spent in different PA intensity levels was calculated and compared between monitors using a mixed model, with a random intercept accounting for multiple observations within each child. The correlation coefficients between the PAW and GT3X+ or GT9X accelerometers were calculated for each PA intensity level using a mixed model with a random intercept, as outlined in Hanlett et al. [53], for the same reason (i.e., multiple observations within each child for each monitor). The bias-corrected 95% confidence intervals for correlation coefficients were estimated using the bootstrapping method, with 200 bootstrap resamples drawn from the observations at the child level.

The agreement of PA estimates between the PAW and GT3X+ (and GT9X) accelerometers was examined using mean absolute percentage error (MAPE) and a modified Bland-Altman method. The mean bias and 95% limits of agreement (LOA) were calculated using a one-way random effects model, where multiple monitoring days are nested within a random factor of subject and the LOA is defined by the true difference between monitors in addition to between- and within-subject random variability. The 95% confidence intervals of LOA were additionally calculated using the method of variance estimates recovery, which has been shown to be superior to a conventional approach using a delta method [54], in order to estimate the maximum limits of upper and lower LOA. Further details of the modified Bland-Altman method, and a SAS macro implementing the calculations of LOA with multiple observations within individuals are available from Zou [54].

In addition, the equivalence test was performed using a two one-sided *t*-test (TOST) approach. By following the guidelines outlined in Dixon et al. [55], two one-sided hypotheses were formulated, where the mean ratio of PA estimates between monitors was compared with the upper and lower limits of 10% equivalence zones (i.e., Ha1: 0.9 < mean ratio; and Ha2: mean ratio < 1.11). Since the primary focus of this study was to examine convergent validity of the PAW relative to the ActiGraph accelerometers, PA estimates obtained from the GT3X+ and GT9X accelerometers were used as references when creating equivalence zones. The selection of 10% equivalence zones was based on previous studies comparing consumer- and research-grade activity monitors [56]. Two variables associated with each hypothesis were created for each monitoring day within each child (i.e., Da1 = PAW − 0.9 × GT3X+ (or GT9X); and Da2 = PAW − 1.1 × GT3X+ (or GT9X)). A mixed model with a random intercept was used to test the mean of each hypothesized variable against zero. Two-sided *p*-value estimated from the model (i.e., intercept parameter) was divided by 2 to obtain the one-sided *p*-value only if the effect was in the hypothesized direction (i.e., positive *t*-value for Da1 and negative *t*-value for Da2). The equivalence of PA estimates of the PAW with the estimates from the GT3X+ and GT9X accelerometers was established when one-sided *p*-values from both tests were less than 0.05 [55]. All data management and statistical analyses were performed using the SAS v9.4 (SAS Institute, Cary, NC, USA), and statistical significance was set at *p* < 0.05.

## 3. Results

**Figure 1:**
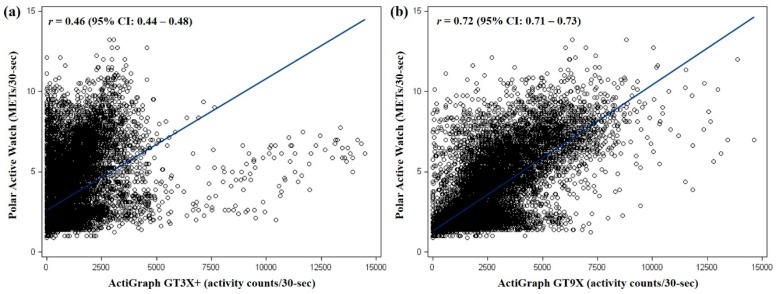
Correlations between metabolic equivalent task (MET) values (per 30-s) of the Polar Active Watch (PAW) and activity counts (per 30-s) of the GT3X+ (**a**) and GT9X (**b**) accelerometers. The diagonal line indicates linear regression line estimated from a mixed model. In the figure (**a**), removing 78 data points with MET values < 7.0 and activity counts > 7000 (at the bottom right in the figure) increased *r* = 0.53 (95% CI = 0.51–0.55).

The scatter plots depicting linear relationships between MET values from the PAW and activity counts from the GT3X+ and GT9X accelerometers are presented in Figure 1. There were positive relationships, where MET values from the PAW were more strongly related with activity counts from the GT9X accelerometer (*r* = 0.72; 95% CI = 0.71–0.73), than those from the GT3X+ accelerometer (*r* = 0.46; 95% CI = 0.45–0.47).

The time spent in PA intensity levels estimated from a mixed model with a random intercept across the monitors are presented in Table 2. The PA estimates from PAW#1 were 19.07 min (95% CI: 16.84–21.30), 19.33 min (95% CI: 17.29–21.37), and 19.02 min (95% CI: 16.45–21.59) for sedentary, LPA, and MVPA, respectively (Table 2). When using PAW#2, significantly lower amount of sedentary (4.57 min; 95% CI = 3.62–5.52) and a larger amount of LPA (32.48 min; 95% CI = 30.23–34.73) were estimated; yet the estimates of MVPA (20.76 min; 95% CI = 18.18–23.34) were not statistically different from PAW#1. PAW#3 yielded statistically different LPA (21.62 min; 95% CI = 19.58–23.66) and MVPA (16.82 min; 95% CI = 14.25–19.39), when compared with the PAW#1 and PAW#2. When compared with the estimates from the GT3X+ accelerometer, the PAW yielded significantly different PA estimates across all intensity levels, regardless of the MET thresholds used. Likewise, PA estimates from the GT9X were statistically different from the estimates of PAW#1 for all intensity levels, but non-significant differences were seen in LPA when compared with PAW#2, and in MVPA when compared with PAW#3, respectively.

**Figure 2:**
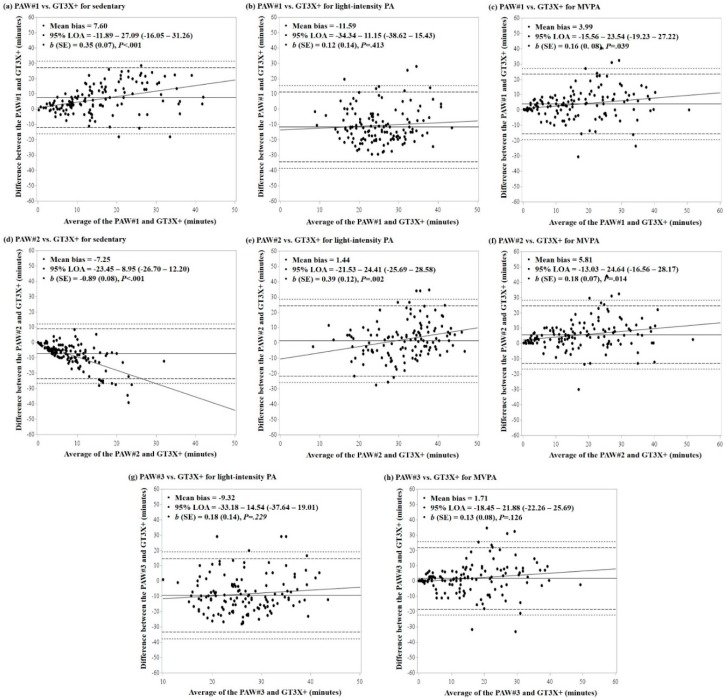
Bland-Altman analysis showing agreement of physical activity (PA) intensity levels between the PAW and GT3X+ accelerometer: (**a**,**b**,**c**) for PAW#1; (**d**,**e**,**f**) for PAW#2; and (**g**,**h**) for PAW#3. The horizontal line at the middle represents the mean bias followed by dotted lines representing 95% of limits of agreement (LOA) and maximum limits of LOA. The diagonal line represents the linear regression line.

**Figure 3:**
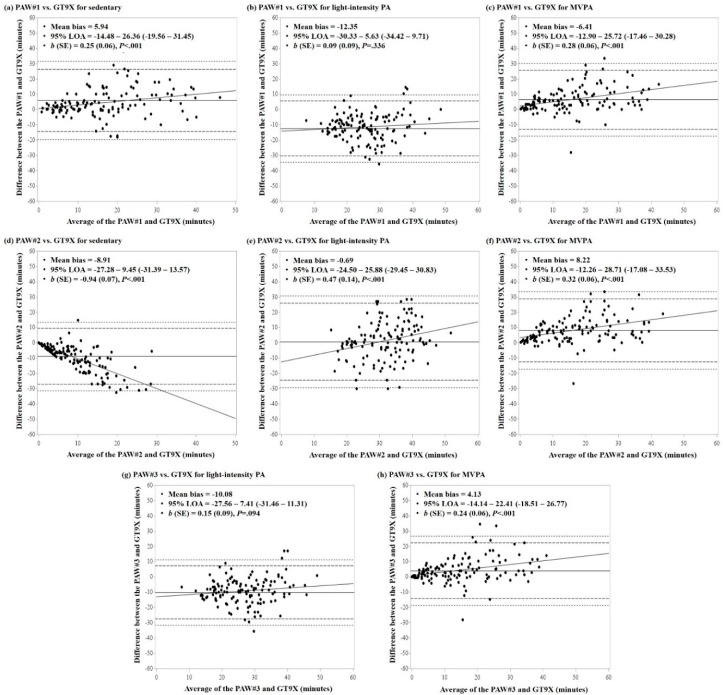
Bland-Altman analysis showing agreement of PA intensity levels between the PAW and GT9X accelerometer: (**a**,**b**,**c**) for PAW#1; (**d**,**e**,**f**) for PAW#2; and (**g**,**h**) for PAW#3. The horizontal line at the middle represents the mean bias followed by dotted lines representing 95% of limits of agreement (LOA) and maximum limits of LOA. The diagonal line represents the linear regression line.

The results of the Bland-Altman analysis showing agreement of PA estimates from the PAW with the GT3X+ and GT9X accelerometers are shown in Figure 2 and Figure 3, respectively. Overall, more than 95% and 98% of data points fell within the 95% LOA and the maximum allowable LOA (i.e., upper and lower limits of the 95% confidence interval of LOA), respectively, across PA intensity levels. Pertaining to the comparison with the GT3X+ accelerometer (Figure 1), the smallest mean bias was observed in LPA minutes from the PAW#2 (mean bias = 1.44 min; 95% LOA: −21.53–24.41) and the largest difference was observed in LPA minutes from the PAW#1 (mean bias = −11.59 min; 95% LOA: −34.34–11.15). The slopes of regression lines showing proportional bias in the agreement were all significant across PA intensity levels and MET thresholds of the PAW, with the exception of LPA in PAW#1 (*b* = 0.12; *P* = 0.413) and PAW#3 (*b* = 0.18; *p* = 0.229), and MVPA in PAW#3 (*b* = 0.13; *p* = 0.126). Similarly, mean bias in comparison with the GT9X accelerometer (Figure 2) was smallest for LPA in PAW#2 (mean bias = −0.69 min; 95% LOA: −24.50–25.88) and largest in the LPA from PAW#1 (mean bias = −12.35 min; 95% LOA: −30.33–5.63). The slopes of regression lines were all significant across PA intensity levels and MET thresholds, with the exception of LPA in PAW#1 (*b* = 0.09; *p* = 0.336) and PAW#3 (*b* = 0.15; *p* = 0.094).

Table 3 presents the correlations, MAPE, and mean ratios of PA estimates from the PAW relative to the GT3X+ and GT9X accelerometers. The correlations of the PAW with the GT3X+ accelerometer ranged between 0.20 and 0.67 for PAW#1, between 0.32 and 0.71 for PAW#2, and between 0.16 and 0.64 for PAW#3, respectively, where the strongest and weakest correlations were found in MVPA and sedentary, respectively, across all MET thresholds of the PAW. When compared with the GT3X+, the largest MAPE was seen in sedentary minutes estimated from PAW#1 (121.68%; 95% CI = 84.87–158.49) and in MVPA estimated from PAW#2 (98.38%; 95% CI = 67.69–129.06) and PAW#3 (69.16%; 95% CI = 47.10–91.22). The lowest MAPE was seen in LPA across all MET thresholds of PAW. The results of the equivalence test using mean ratios based on the TOST approach demonstrated non-equivalence of PA estimates of the PAW, at 10% of the equivalence zones of the GT3X+ accelerometer. Similarly, when compared to the GT9X accelerometer, the weakest correlations were found in LPA and strongest correlations were found in MVPA across all MET thresholds of the PAW. The MAPE was largest in MVPA and smallest in LPA across all MET thresholds of PAW. The significant equivalence was observed in LPA estimated from PAW#2 (mean ratio: 1.11; 95% CI = 1.01–1.20), but no other equivalency was found relative to 10% of the equivalence zone of the GT9X accelerometer.

## 4. Discussion

The purpose of this study was to examine the convergent validity of the PAW, a consumer-grade PA monitor, for the assessment of PA intensity levels in children, against research-grade and waist-worn GT3X+ and wrist-worn GT9X accelerometers. The three sets of MET thresholds were applied to the PAW for the estimation of PA intensity levels, and PA estimates were compared with the estimates obtained from the GT3X+ using Evenson’s cut-points and the GT9X using Chandler’s cut-points. Our findings demonstrated that PA estimates from the PAW were moderately to highly correlated with the estimates from the GT3X+ and GT9X accelerometers for sedentary (*r* ranges 0.45–0.66) and MVPA (*r* ranges 0.64–0.75), whereas weak to moderate correlations were found for LPA (*r* ranges 0.16–0.54). PA estimates obtained from the PAW generally showed greater correlations with the estimates from the GT9X (*r* ranges 0.20–0.75) than the GT3X+ (*r* ranges 0.16–0.71) for each intensity level. In particular, greater correlations were found for the PAW using standard MET thresholds (PAW#2) with the GT3X accelerometer (*r* ranges 0.32–0.71), and PAW using adjusted MET thresholds (PAW#3) showed greater correlations with the GT9X accelerometer (*r* ranges 0.66–0.75).

The convergence between monitors is established when PA estimates from the monitors are sufficiently correlated; however, there is little consensus about the optimal correlation coefficient to determine the level of convergent validity. The rules of thumb that have been widely applied in the literature are to interpret a correlation between 0.0 and <0.25 as a weak relationship, ≥0.25–<0.50 as a moderate relationship, ≥0.50–<0.75 as a strong relationship, and ≥0.75 as a very strong relationship. However, it is also suggested that the correlation coefficients be interpreted relative to common practice in a field [57]. As previously noted, there is currently little evidence available in the literature regarding the convergent validity of the PAW in comparison with a research-grade PA monitor; however, there are several studies available that examined the convergent validity of different consumer-grade PA monitors against the ActiGraph accelerometers. One study compared the waist-worn Fitbit Zip (Fitbit Inc., San Francisco, CA, USA) with the waist-worn GT3X+ accelerometer in children, particularly using the Evenson’s cut-points, where interpreted correlations ranged from 0.24–0.72 for MVPA minutes as weak to moderate and 0.57–0.87 for sedentary minutes as moderate to strong correlations [44]. Among the adult population, Gomersall et al. [58] reported correlations of MVPA minutes estimated from the Fitbit One and Jawbone Up (Jawbone Inc., San Francisco, CA, USA) to be 0.80 and 0.72, respectively, compared to the GT3X accelerometer, and interpreted them as strong correlations [58]. Another study that compared six consumer-grade PA monitors with the GT3X accelerometer demonstrated correlations ranging between 0.52 and 0.91 for MVPA minutes and interpreted them as weak to strong correlations. Although there are some discrepancies in interpreting the correlations within the contexts of convergent validity of consumer-grade PA monitors in the literature, it is also suggested that *r* ≥ 0.70 is recommended to claim convergent validity, and *r* < 0.50 should be avoided [38]. In this regard, our findings may demonstrate moderate (*r* ≥ 0.50) convergent validity of the PAW, particularly using the Polar-defined (PAW#1) and adjusted MET thresholds (PAW#3), for the estimation of sedentary minutes against the GT3X+ and GT9X accelerometers. Pertaining to MVPA minutes, the PAW, regardless of which MET thresholds were used, showed at least moderate convergent validity relative to the GT3X+ and greater convergent validity was demonstrated when compared with the GT9X accelerometer. However, there was weak convergent validity for the estimation of LPA minutes. 

Aside from interpretation of correlation coefficients within the context of convergent validity of consumer-grade monitors, our findings can be compared with Mossea et al. [44] who examined the validity of the Fitbit Zip in relation to the GT3X+ accelerometer in a school environment. In particular, their findings partly in contrast with our results by showing greater correlations in MVPA than sedentary minutes; but some similarities were also observed. In their study, the highest correlation of 0.72 for MVPA was reported during a physical education lesson, followed by a correlation of 0.56 observed at recess, which were comparable with the correlations that we observed between the PAW and GT3X+ accelerometer for MVPA minutes (*r* ranges 0.67–0.71). Whereas the correlations for sedentary minutes during physical education and recess time were reported as 0.85 and 0.87, respectively; these were higher than the correlations we observed for sedentary minutes against the GT3X+ accelerometer (*r* ranges 0.48–0.65). Although it is still difficult to directly compare the results between the studies due to the fundamental differences between the monitors (e.g., uniaxial vs. triaxial accelerometer sensors for the PAW and Fitbit Zip, respectively), this may indicate that convergent validity of the PAW for the estimation of MVPA minutes is comparable with other consumer-grade monitors, but not for sedentary minutes. 

The correlation is a primary method for evaluating the convergent validity; however, it is not sensitive for detecting systematic difference in mean values between the monitors, as well as variability of differences within the group. In the present study, we found that, in general, the mean of PA estimates from the PAW were not statistically equivalent with the estimates from the GT3X+ and GT9X accelerometers, regardless of which MET thresholds were used for the PAW. Although more than 95% of the data points were within the observed LOAs in the Bland-Altman analyses, a relatively wide range of LOA indicates large variability in differences within the group. We further observed significant proportional bias where mean differences tended to increase with increasing average estimates between the monitors. Considering that this study focused on the 60-min of afterschool programs, it should be noted that longer measurement periods may likely increase differences in PA estimates between the PAW and GT3X+/GT9X accelerometers, as well as within-group variability in differences.

Meanwhile, present findings also indicated that the PAW consistently overestimated time spent in MVPA relative to the GT3X+ and GT9X accelerometers, with the largest MAPE (168.56%) observed between PAW#2 and the GT9X accelerometer, despite the strong correlation observed (*r* = 0.72). In contrast, MAPE was consistently lower in comparing LPA minutes between the monitors across the MET threshold conditions of the PAW. The smallest MAPE was observed when comparing PAW#2 and GT3X+ (30.50%), where the lowest correlation (*r* = 0.16) was also calculated. Conceptually, the correlation is a measure of the linear relationship between the monitors showing consistency of the relative position of the same participants between the monitors; whereas MAPE is a measure of absolute error in PA estimates between the monitors. These results imply that MVPA minutes obtained from the PAW may have limited comparability with the estimates from the GT3X+ and GT9X accelerometers at group level; however, the relative position of the participants within the group may be comparable between the monitors. Whereas, less difference may be expected when comparing the group mean of LPA minutes between the monitors, large unknown random errors may reduce the comparability of concordant changes in LPA minutes within the group.

The underlying mechanisms explaining the observed systematic and proportional biases could not be clearly elucidated in the present study, but it should be noted that selection of the MET thresholds proportionally influenced PA estimates from the PAW. For the children, the use of standard MET thresholds (<1.5 METs for sedentary and ≥3 METs for MVPA) for the estimation of PA intensity levels has been concerned with whether the rate of energy expenditure is based on a standard resting metabolic rate (RMR) for adults (3.5 mL/kg/min), rather than for children, whose RMR can be up to 6 mL/kg/min [59]. As an alternative, the thresholds with <2.0 METs for sedentary and ≥4 METs for MVPA have been proposed as adjusted MET thresholds if the standard adult’s RMR is used [46,60]. As previously noted, it is currently unclear if the PAW produces the MET values after accounting for child-specific RMR. The Polar standard MET thresholds define sedentary as >2.0 METs and MVPA as ≥3.5 METs, which are 0.5 METs greater than standard MET thresholds and 0.5 METs lower than adjusted MET thresholds for MVPA, resulting in significantly different PA estimates when compared with PA estimates obtained using standard and adjusted MET thresholds. The Evenson’s cut-points were developed based on the ten semi-structured activities ranging from sedentary to vigorous-intensity PA, where the maximum MET value of sedentary activities and minimum MET value of MVPA activities were about 1.3 and 3.7 METs, respectively, after accounting for child-specific RMR. In this regard, our results that showed less mean difference in MVPA minutes when using adjusted MET thresholds (PAW#3) with the GT3X+ accelerometer may indicate that child-specific RMR is not accounted for when estimating MET values from the PAW. However, inconsistent findings were found where sedentary and LPA minutes were over and underestimated by PAW#3 relative to the GT3X+ accelerometer, respectively, which may also indicate that the PAW is not sensitive to discriminate activities between sedentary and LPA levels when compared to the GT3X accelerometer.

It is widely acknowledged that the placement of the activity monitor may alter measured PA outcomes. In particular, a wrist-worn PA monitor is generally known to be more sensitive to upper-body locomotor movements [61,62] and we expected high comparability between the PAW and GT9X accelerometer, as both monitors were placed on the same non-dominant wrist. In the present study, there was stronger correlation (*r* = 0.72) of MET values from the PAW with the activity counts from the wrist-worn GT9X accelerometer, compared to the waist-worn GT3X accelerometer (*r* = 0.46). However, as previously discussed, the amounts of PA measured by the PAW were not comparable to the GT9X accelerometer. On the other hand, there was no systematic mean bias between the GT3X and GT9X accelerometers (*p* > 0.05; the results are not shown in the table), with strong correlations across all intensity levels between the monitors (*r* ≥ 0.87). This may support the notion that appropriate calibration of raw accelerometry data obtained from waist- and wrist-worn activity tracker would result in comparable PA estimates [63,64]. In this regard, the lack of comparability of the PAW with the GT9X, as well as the GT3X, accelerometers may be attributed to unknown measurement errors introduced when calibrating raw accelerations into the MET values. Currently, most companies manufacturing consumer-grade PA monitors do not provide access to raw acceleration data nor their proprietary algorithms. For the PAW, it is likely that the required input parameters including age, sex, height, and weight are accounted for when estimating MET from the Polar software; however, the detailed parameters and proprietary algorithms are not publicly available. Thus, it is difficult to elucidate where exactly the observed discrepancies arose in the current study. In addition, the proprietary algorithms of most companies are often changed over time as new functions or results from their own discrete research are added, affecting intra-monitor reliability, as well as comparability with other consumer- and research-grade PA monitors [65]. Therefore, we echo the recommendation of Evenson et al. [19] suggesting that these companies consider revealing their proprietary algorithms, at least to some extent (e.g., calibration methods, input parameters, and research outputs), which can potentially advance measurement practice of both consumer- and research-grade PA monitors.

There are several limitations that should be considered when interpreting the present findings. First, our study did not have a criterion measure of energy expenditure, so we were unable to draw any conclusions on the criterion validity of the PAW. Additionally, as previously noted, the Evenson’s and Chandler’s cut-points were developed using different models of ActiGraph accelerometers. Although we enabled the low frequency extension filter during post data processing to reduce possible bias for using different models of ActiGraph accelerometers, where recent studies have shown high comparability of activity counts between the GT3X+ and GT9X accelerometers, both monitors cannot be considered criterion measures of PA. Furthermore, the Evenson’s and Chandler’s cut-points were converted to 30-s cut-points which might introduce errors in estimating time spent in PA intensity levels from the GT3X+ and GT9X accelerometers. Collectively, our findings should be interpreted within the context of convergent validity of the PAW in comparison with the waist-worn GT3X+ and the wrist-worn GT9X accelerometers using Evenson’s and Chandler’s cut-points, respectively. Future work should examine the accuracy of the PAW against a criterion measure of energy expenditure, such as indirect calorimetry. In addition, the MET threshold for LPA in PAW#3 was 2.00–3.99 METs based on a recent study that reported higher accuracy of <2.0 METs for the classification of sedentary activities in children when child-specific RMR is not accounted [47]. However, the MET thresholds of LPA used in Evenson’s original calibration study [40] was about 1.5–3.99 METs, and this discrepancy in MET thresholds for LPA might introduce the errors when comparing LPA minutes between PAW#3 and GT3X+ accelerometer. Lastly, PA observations were exclusively taken from the 80-min school-based afterschool program setting, and we did not collect the specific activities that occurred during the program. Although the observations were taken across multiple schooldays to increase total observation time per individual and the types of activity that took place included all intensity levels ranging from sedentary to vigorous-intensity, we suggest that future research should utilize longer monitoring times in free-living settings or include a wide range of unstructured and structured activities in a controlled laboratory setting.

## 5. Conclusions

We attempted to examine the convergent validity of the PAW for the assessment of PA in children compared to the waist-worn GT3X+ and wrist-worn GT9X accelerometers. Overall, the results indicated a weak convergent validity of the PAW for the assessment of LPA minutes, but moderate convergent validity for the estimation of sedentary and MVPA minutes, relative to the GT3X+ and GT9X accelerometers, particularly when applying the Polar-defined or adjusted MET thresholds. There were significant mean differences in PA estimates between the monitors, with less discrepancy observed in MVPA minutes when using the adjusted MET thresholds. Collectively, the data suggested that within-group comparison of relative levels of sedentary and MVPA minutes estimated from the PAW could be comparable to those of the GT3X+ and GT9X accelerometers. However, between-group comparisons of PA estimates obtained from different monitors is not recommended due to the large uncertainties regarding their comparability at group-level, particularly when using the Polar-defined MET thresholds. Such uncertainties can be possibly attenuated when applying the adjusted MET thresholds for the estimation of PA levels from the PAW. Given the potential of PAW as a means of PA assessment or as an intervention component among children, across both research and practical applications, further research is needed to examine the criterion validity of the PAW in children.

## Figures and Tables

**Table 1:** Descriptive characteristics of the participants.

	Total	Boys	Girls
*n*	51	18	33
Age (years)	10.30 (0.91)	10.33 (0.91)	10.30 (0.92)
Race/Ethnicity (*n*, %)			
Non-Hispanic Black	31 (60.78%)	10 (55.56%)	21 (63.63%)
Hispanic	12 (23.53%)	6 (33.33%)	6 (18.18%)
Others	8 (15.69%)	2 (11.11%)	6 (18.18%)
Height (cm)	140.18 (8.42)	138.39 (7.36)	141.97 (8.69)
Weight (kg)	41.17 (11.02)	37.41 (7.67)	44.92 (11.63)
Body mass index (kg/m^2^)	21.38 (4.24)	19.53 (3.39)	22.29 (4.36)
Monitoring days ^a^	3.0 (2.0–4.0)	3.0 (3.0–4.0)	3.0 (2.0–4.0)

Values are mean (standard deviations) unless otherwise specified. ^a^ number of days where physical activity data were recorded during afterschool hours—values are median (interquartile range).

**Table 2:** Average time spent in different physical activity intensity levels estimated from PAW, GT3X+ and GT9X accelerometers ^a^.

	Sedentary	Light	MVPA
Polar Active Watch (PAW)			
MET thresholds#1 (PAW#1)	19.07	19.33	19.02
	(16.84–21.30) ^†^	(17.29–21.37) ^†,^^‡^	(16.45–21.59) ^‡^
MET thresholds#2 (PAW#2)	4.57	32.48	20.76
	(3.62–5.52) *	(30.23–34.73) *^,^^‡^	(18.18–23.34) ^‡^
MET thresholds#3 (PAW#3) ^b^	-	21.62	16.82
		(19.58–23.66) *^,†^	(14.25–19.39) *^,†^
ActiGraph GT3X+	12.34	30.46	15.03
	(10.69–13.99) *^,†,^^‡^	(28.56–32.35) *^,†,^^‡^	(13.01–17.06) *^,†^
ActiGraph GT9X	14.16	31.22	12.39
	(12.13–16.19) *^,†,^^‡^	(29.29–33.14) *^,^^‡^	(10.22–14.56) *^,†,^^‡^

^a^ Values are presented as mean minutes and 95% confidence interval estimated from a mixed model with a random intercept, after accounting for multiple observations within each child; ^b^ sedentary MET threshold in PAW#3 (<2 MET) is identical to the PAW#1, and thus the results are omitted. * significantly different with the PAW#1; ^†^ significantly different with the PAW#2; ^‡^ significantly different with the PAW#3.

**Table 3:** Correlations, Mean Absolute Percentage Error (MAPE), and Mean Ratios for Equivalence Tests between PAW, GT3X+, and GT9X Accelerometers ^a^.

	Polar Active Watch (PAW) MET Thresholds#1 (PAW#1)			Polar Active Watch (PAW) MET Thresholds#2 (PAW#2)			Polar Active Watch (PAW) MET Thresholds#3 (PAW#3)		
	Correlation ^b^	MAPE ^c^	Mean Ratio ^d^	Correlation ^b^	MAPE ^c^	Mean Ratio ^d^	Correlation ^b^	MAPE ^c^	Mean Ratio ^d^
ActiGraph GT3X+									
Sedentary	0.65	121.68	2.06	0.48	69.92	0.44	-	-	-
	(0.54–0.76)	(84.87–158.49)	(1.68–2.44)	(0.28–0.69)	(63.39–76.44)	(0.33–0.57)			
Light PA	0.20	47.00	0.72	0.32	35.50	1.15	0.16	30.50	0.77
	(−0.03–0.43)	(40.44–53.57)	(0.63–0.80)	(0.13–0.52)	(28.39–42.61)	(1.06–1.24)	(−0.06–0.39)	(34.20–44.82)	(0.69–0.86)
MVPA	0.67	88.34	1.69	0.71	98.38	1.88	0.64	69.16	1.40
	(0.54–0.80)	(60.24–116.44)	(1.38–2.00)	(0.58–0.83)	(67.69–129.06)	(1.56–2.19)	(0.49–0.78)	(47.10–91.22)	(1.15–1.65)
ActiGraph GT9X									
Sedentary	0.66	122.73	2.07	0.45	79.84	0.51	-	-	-
	(0.49–0.82)	(53.9–191.57)	(1.36–2.78)	(0.20–0.70)	(55.21–104.46)	(0.20–0.82)			
Light PA	0.49	40.94	0.62	0.20	34.48	1.11	0.54	35.06	0.69
	(0.31–0.68)	(36.22–45.65)	(0.56–0.69)	(0.00–0.40)	(28.00–40.96)	(1.01–1.20) *	(0.37–0.72)	(30.70–39.41)	(0.63–0.76)
MVPA	0.74	128.04	2.20	0.72	168.56	2.63	0.75	94.33	1.80
	(0.59–0.90)	(84.08–172.00)	(1.75–2.66)	(0.57–0.87)	(97.48–239.64)	(1.91–3.35)	(0.61–0.90)	(57.50–131.16)	(1.42–2.19)

^a^ sedentary MET threshold in PAW#3 (<2 MET) is identical to PAW#1, and thus the results are omitted; ^b^ correlation coefficients were estimated from a mixed model with a random intercept [53] and bias-corrected 95% confidence intervals were estimated using the bootstrapping method; ^c^ values represent mean absolute percentage error (MAPE, %) and 95% confidence intervals in parentheses; ^d^ values represent mean ratio of PA estimates of the PAW over the estimates from the GT3X+ or GT9X accelerometers and 95% confidence intervals in parentheses. * significantly equivalent with the PAW#1 based on two one-sided *t*-tests (TOST) equivalence test.
