# Erratum: Kim, Y., et al. Comparison of Polar Active Watch and Waist- and Wrist-Worn ActiGraph Accelerometers for Measuring Children’s Physical Activity Levels during Unstructured Afterschool Programs. *Int. J. Environ. Res. Public Health* 2018, *15*, 2268

**DOI:** 10.3390/ijerph16030446

**Published:** 2019-02-03

**Authors:** Youngdeok Kim, Marc Lochbaum

**Affiliations:** 1 Department of Kinesiology and Sport Management, Texas Tech University, Lubbock, TX 79409, USA; marc.lochbaum@ttu.edu; 2 The Academy of Education, Vytautas Magnus University, Kaunas LT-44248, Lithuania

Due to an error during production, the Chandler’s cut-points at the 30-sec epoch length [1] were incorrectly presented in the published paper [2]. The corrected cut-points are as follows:

## Incorrect cut-points:

Sedentary (<805), Light-intensity physical activity (805–2645), and moderate- and vigorous-intensity physical activity (≥2646).

## Correct cut-points:

Sedentary (<966), Light-intensity physical activity (966–3174), and moderate- and vigorous-intensity physical activity (≥3175).

These changes do not influence the results, discussion, or conclusions. The authors would like to apologize for any inconvenience caused to the readers by this error.
